# Fucoxanthin: A Marine Carotenoid Exerting Anti-Cancer Effects by Affecting Multiple Mechanisms 

**DOI:** 10.3390/md11125130

**Published:** 2013-12-16

**Authors:** Sangeetha Ravi Kumar, Masashi Hosokawa, Kazuo Miyashita

**Affiliations:** Faculty of Fisheries Sciences, Hokkaido University, 3-1-1, Minato Cho, Hakodate, Hokkaido 041-8611, Japan; E-Mails: hoso@fish.hokudai.ac.jp (M.H.); kmiya@fish.hokudai.ac.jp (K.M.)

**Keywords:** anti-proliferative, apoptosis, carotenoid, cell cycle arrest, fucoxanthin

## Abstract

Fucoxanthin is a marine carotenoid exhibiting several health benefits. The anti-cancer effect of fucoxanthin and its deacetylated metabolite, fucoxanthinol, is well documented. In view of its potent anti-carcinogenic activity, the need to understand the underlying mechanisms has gained prominence. Towards achieving this goal, several researchers have carried out studies in various cell lines and *in vivo* and have deciphered that fucoxanthin exerts its anti-proliferative and cancer preventing influence via different molecules and pathways including the Bcl-2 proteins, MAPK, NFκB, Caspases, GADD45, and several other molecules that are involved in either cell cycle arrest, apoptosis, or metastasis. Thus, in addition to decreasing the frequency of occurrence and growth of tumours, fucoxanthin has a cytotoxic effect on cancer cells. Some studies show that this effect is selective, *i.e*., fucoxanthin has the capability to target cancer cells only, leaving normal physiological cells unaffected/less affected. Hence, fucoxanthin and its metabolites show great promise as chemotherapeutic agents in cancer.

## 1. Introduction

**Figure 1:**
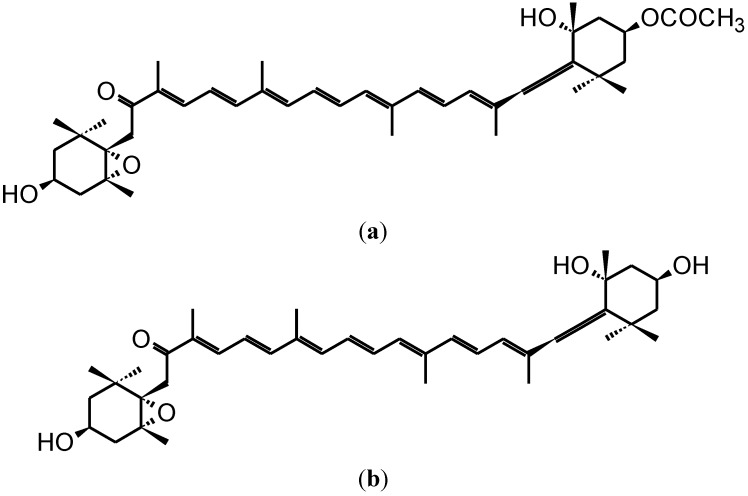
Chemical structure of fucoxanthin (**a**) and its deacetylated metabolite, fucoxanthinol (**b**).

**Figure 2:**
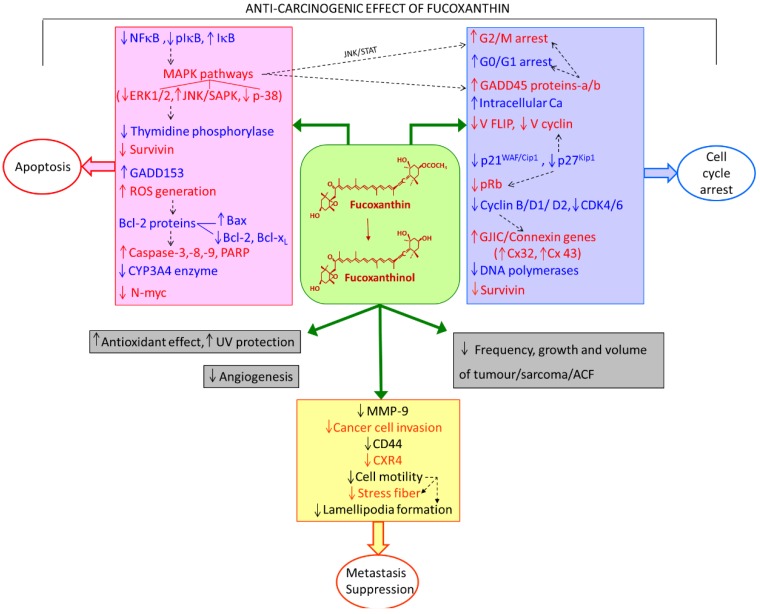
Factors (molecules and mechanisms) regulated by fucoxanthin, resulting in its anti-carcinogenic effects. Dashed lines indicate inter-relation/inter-effects between the factors; up and down arrows indicate up- and down-regulation (by fucoxanthin/fucoxanthinol), respectively.

Fucoxanthin is a marine carotenoid found in numerous classes of microalgae (e.g., bacillariophytes, bolidophytes, chrysophytes, silicoflagellates, pinguiophytes) and brown macroalgae (phaeophytes) [1,2]. The chemical structure of fucoxanthin includes an allenic bond and oxygenic functional groups, such as hydroxyl, epoxy, carbonyl, and carboxyl groups in addition to its polyene chain (Figure 1). It is speculated that one of the reasons for the longevity of certain populations is the regular consumption of seaweeds including brown algae [3], which are known to be the major sources of fucoxanthin. Studies on brown seaweeds rich in fucoxanthin have revealed their anti-cancer effects [4,5,6,7,8]. Hence, the effect of fucoxanthin on cancer is of interest and has been studied by several researchers. The unanimous result of the studies on fucoxanthin in cancer has established that fucoxanthin performs a protective role and exhibits anti-proliferative behavior in various types of cancer. Recently, Gagez *et al*. [1] have reviewed the biological activities of epoxycarotenoids including fucoxanthin in cancer cells grown *in vitro* and described the various cellular targets of fucoxanthin. With the establishment of the anti-carcinogenic property of fucoxanthin, it was important to understand the mechanism by which it exerted its effect in cells. With this goal in mind, several researchers have been trying to elucidate the molecules and pathways that can be modulated and regulated by fucoxanthin. Mechanistic studies by various researchers have shown that fucoxanthin can affect many cellular processes, and so far have failed to establish a single primary mechanism of action. The objective of this review is to summarize the effect of fucoxanthin in cancer and the underlying mechanisms that have been elucidated in reported studies. The various mechanisms discussed further in this review are shown in Figure 2.

## 2. Anti-Carcinogenic Effects of Fucoxanthin

### 2.1. Decreased Incidence of Tumors

Nishino [9] has reported suppression of skin tumor formation as well as *N*-ethyl-*N*′-nitro-*N*-nitrosoguanidine (ENNG)-induced mouse duodenal carcinogenesis. In addition to the decrease in the percentage of tumor bearing mice, the mean number of tumors per mouse was also significantly lower in fucoxanthin fed mice. A decrease in the percentage of tumor-bearing mice as well as a decrease in the number of tumors induced per mouse by ENNG in the fucoxanthin group was also observed by Okuzumi and co-workers [10,11]. Administration of fucoxanthin resulted in significant decrease of sarcoma weight in mice [12]. Das *et al*. [4] and Kim *et al*. [13] have observed that aberrant crypt foci (ACF) formation in rats was decreased by fucoxanthin. The *in vivo* study by Ishikawa *et al*. [14] revealed unchanged tumor incidence but delayed tumor growth and decreased tumor volume with the deacetylated metabolite of fucoxanthin, namely fucoxanthinol. In addition, in contrast to the untreated tumors, abundant apoptotic cells were observed in tumors from the fucoxanthinol treated group. Fucoxanthin also suppressed the growth of B16F10 melanoma in Balb/c mice [15].

### 2.2. Antioxidant Effect of Fucoxanthin

Several reports on the potent antioxidant property of fucoxanthin and its metabolites are available [16,17,18,19,20]. While its antioxidant property was initially thought to be the main reason behind its anti-carcinogenic effect, it is now established that the realm of fucoxanthin’s effect is wider and involves several other biological processes as well. Moreover, some studies have reported the pro-oxidant effect of fucoxanthin on cancer cells with the production of free radicals and have proposed this to be one of the mechanisms by which it protects against cancer cells. Kotake-Nara *et al*. [21] have hypothesized that the prooxidant actions of fucoxanthin and other carotenoids used in their study may be the reason for the induction of apoptosis in cancer cells. However, in their subsequent study [22], they have found similar apoptotic activity of fucoxanthin in promyelocytic leukemia cell lines but from their results in H_2_O_2_ resistant cell lines, they have concluded that reactive oxygen species (ROS) is not the mainstream pathway for apoptosis caused. Contradictory to this, Kim *et al*. [23] have observed the growth inhibition in leukemia cell lines by fucoxanthin and have attributed it to radical oxygen species (ROS) generation by fucoxanthin that leads to apoptosis. They observed increased production of H_2_O_2_ and O_2_^−^ as a result of treatment with fucoxanthin along with accumulation of cells containing sub G1 DNA content (indicating cell cycle arrest in G1 stage). On co-treatment with a commercial antioxidant (NAC), the number of apoptotic bodies and DNA fragmentation of cells was decreased, attributing the apoptotic effect of fucoxanthin to ROS generated. Thus, they have concluded that cytotoxic effect of fucoxanthin is by ROS generation that triggers apoptosis in HL-60 cells, which is contrary to the results of Kotake-Nara *et al*. [22]. Shimoda *et al*. [24] have reported the anti-pigmentation effect of fucoxanthin and suppression of melanogenesis in melanoma and tyrosinase activity in UV-irradiated guinea pigs. Heo and Jeon [25] have reported the protective effect of fucoxanthin on exposure of human fibroblasts to UVB exposure and attributed the protective effects to the antioxidant activity of fucoxanthin.

### 2.3. Cell Viability/Anti-Proliferation of Cells

The effect of fucoxanthin on cell viability in cancer cells such as GOTO, HL-60, Caco-2, HepG-2, Neuro2a, DU145, PEL, PC-3, HeLa, H1299, HT-29, DLD-1 cells, and *in vivo* has been explored by many researchers [10,14,21,22,26,27,28,29,30,31,32,33,34]. Liu *et al*. [29] have reported the anti-proliferative effect of fucoxanthin against SK-Hep-1 (human hepatoma) cells and BNL CL.2 (murine embryonic liver) cells. They have reported a strong correlation between fucoxanthin concentration and anti-proliferative effect on SK-Hep-1 cells at 24 h. However, the suppressive effect was similar for concentrations >1 µM after 48 h. Fucoxanthin, however, was found to facilitate the growth of BNL CL.2 cells until 24 h after which there was a slight decrease in proliferation at 48 h indicating that fucoxanthin was selectively more effective against the SK-Hep-1 cells. In a separate study, Hosokawa *et al*. [27] compared the effects of fucoxanthin and other carotenoids such as β-carotene and astaxanthin on colon cancer cell lines (Caco-2, HT-29, and DLD-1). Cell viability was found to be significantly decreased with fucoxanthin as compared to the other carotenoids and the Caco-2 cell line was found to be most sensitive to the action of fucoxanthin.

Yamamoto *et al*. [31] have concluded that the effect of fucoxanthinol (deacetylated metabolite of fucoxanthin) was more potent than fucoxanthin and PEL cells were more susceptible to the effects of fucoxanthin and fucoxanthinol than HeLa cells. Kotake-Nara *et al*. [21] have reported a dose dependent reduction in cell viability in prostate cancer cell lines exposed to fucoxanthin along with morphological changes such as rounding up, detachment and reduction in cell volume and apoptotic bodies. The DNA fragmentation observed in cells treated with fucoxanthin suggested that apoptosis was the cause of suppression of cell viability. In a separate study on the effect of neoxanthin and fucoxanthin on PC-3 prostate cancer cells, Kotake-Nara *et al*. [35] have reported decreased cell viability, rounding up, reduced cell volume, chromatin condensation, nuclei fragmentation, formation of apoptotic bodies in addition to the apoptotic DNA ladder indicating apoptosis in the cells. In a recent study by Ganesan *et al*. [26] on 11 carotenoids, two marine carotenoids, siphonaxanthin and fucoxanthin were found to possess potent growth inhibitory and apoptosis inducing effect in HL-60 leukemia cells. Cell viability was reduced with fucoxanthin treatment and apoptosis was characterized by DNA fragmentation and chromatin condensation. Jaswir *et al*. [28] have reported the dose dependent growth inhibition of H1299 (lung cancer) cells and morphological changes, such as decrease in cell size and nuclear condensation.

Thus, fucoxanthin was found to have a significant effect on cell viability and anti-proliferative effect and in several studies the potency was different for different cell types/lines. In addition, in several studies, the normal cells were unaffected/less affected than cancer cells indicating differential effect of fucoxanthin and focused targeting of cancer cells [29,31,34,36,37,38]. However, it is important to note that due to different growth rates of cancer and normal cells, attention to experimental detail is a very important factor while drawing conclusions. Therefore, very rigorous methods are essential to prove cancer selectivity over normal cells in experimental studies. Another interesting finding in a few of the studies was the greater potency of the deacetylated metabolite of fucoxanthin (fucoxanthinol). Fucoxanthinol is known to be the major fucoxanthin metabolite [39,40,41]. Dietary fucoxanthin is hydrolyzed to fucoxanthinol in the gastrointestinal tract by digestive enzymes such as lipase and cholesterol esterase. Fucoxanthinol was detectable at 0.8 nM in human plasma after a daily intake (6 g dry weight containing 6.1 mg (9.26 mmol) of fucoxanthin) of cooked edible brown seaweed, *Undaria pinnatifida* (Wakame), for one week [41]. In another study, Hashimoto *et al*. [42] have reported 7.6 nM of fucoxanthinol after 24 h, on administration of kombu extract containing 31 mg of fucoxanthin in human subjects.

### 2.4. Cell Cycle Arrest

The arrest of the cell cycle in the G_0_/G_1_ stage by fucoxanthin has been observed in many studies involving different cell lines [10,14,15,31,32,43,44,45,46] while Yu *et al*. [47] have observed cell cycle arrest in G_2_/M phase. Muthuirullappan and Francis [48] have attempted to review some of these studies recently and explored the possibility of a nano-suspension formulation for fucoxanthin. Liu *et al*. [29] have reported the anti-proliferative effect of fucoxanthin with enhanced gap junction intracellular communication (GJIC) and increased intracellular calcium ions. They have suggested that the enhanced expression of connexin genes and GJIC may increase intracellular calcium levels resulting in cell cycle arrest and apoptosis. Accumulation of cells in the G_0_-G_1_ phase with a significant decrease in cells in the S phase, indicating a block in the progression of the cells to S phase from the G_0_-G_1_ phase, resulting in inhibition of proliferation of the cells has been reported in GOTO cell line [10]. Fucoxanthin arrested cell growth in the G_1_ stage and this was accompanied by alteration in the expression of more than 50 genes in HEPG2 cells [32]. In addition to the GADD45 gene expression, the expression of other growth related genes such as PIM 1, IFRDI, p21, and p27 was also increased. Ishikawa *et al*. [14] have found decreased expression of cyclin D1, cyclin D2, CDK4, CDK6, and cIAP2 on fucoxanthin treatment in leukemia. Kim *et al*. [15] have observed inhibited cell growth, morphological changes and apoptosis in melanoma cells (B16F10) on exposure to fucoxanthin. This was accompanied by a sub G_1_ peak along with concentration of cells in G_0_/G_1_ phase and their decrease in the S and G_2_/M phases. In addition, pRb, cyclin D1, cyclin D2, and CDK4 levels were decreased along with increased p^15INK4B^, p^27KIP1^ levels. Murakami *et al*. [49] have reported the inhibition of DNA polymerases, especially pol α activity at lower concentrations (79 µM) and pol β as well at higher concentrations (100 µM) by fucoxanthin, *in vitro*.

Das *et al*. [45] have observed continuous cell cycle arrest at G_0_/G_1_ phase at lower concentrations of fucoxanthin (25 µM), followed by apoptosis at high concentrations (>50 µM) with increased cells in sub G_1_ phase (index of apoptotic DNA fragmentation) and fragmentation of nuclei. Low and high concentrations of fucoxanthin up regulated the protein and mRNA levels of p21^WAF1/Cip1^ followed by increased levels of pRb (retinoblastoma protein), while high levels up regulated p27^Kip1^ as well (cdk inhibitory proteins) leading to the conclusion that fucoxanthin-induced G_0_/G_1_ cell arrest is mediated by the up regulation of p21^WAF1/Cip1^. They have speculated that the apoptosis observed at higher concentration may be due to partial conversion of fucoxanthin to its metabolites such as fucoxanthinol. From their results they have concluded that p21^WAF1/Cip1^ is important for the cell cycle arrest while p27^Kip1^ regulation may be a means for pro-apoptotic effect of fucoxanthin. Reduction in the phosphorylation of pRb protein, which is a regulator of cell cycle progression and down-regulation of other cell cycle regulatory proteins like cyclin D2, CDK4, CDK6, and c-Myc was reported by Yamomoto *et al*. [28]. A decrease was observed in the pRb levels while the total Rb protein concentration remained constant and the activity of cyclin D/cdk4 was decreased by fucoxanthin in the study of Das *et al*. [46]. Fucoxanthin treatment resulted in decreased protein and mRNA levels of cyclins D1 and D3 while protein level of cdk4 was unaffected at 12 h. The protein levels of p27^Kip1^ p21^Waf/Cip1^, p57, and p16 were unchanged over this period indicating that inhibition of cyclin D/cdk4 activity by fucoxanthin was brought about by suppressing the levels of proteolysis and transcription of cyclin D. An increase in proteosomal activity was observed after 12 h of fucoxanthin treatment. Thus, they have suggested that fucoxanthin induced cell cycle arrest by suppression of cyclin D by proteosomal degradation and transcriptional repression. They have speculated that the decreased cyclin D expression may be due to change in the GADD45A expression.

### 2.5. Apoptosis: Cytotoxic Effect

Apoptosis of cancer cells is a promising method to control and treat cancer. In this regard, the apoptotic effect of fucoxanthin is of interest and has been studied by several researchers. Hosokawa *et al*. [33] have reported DNA fragmentation and the DNA ladder characteristic of apoptosis in HL-60 cells treated with fucoxanthin. In a separate study, fucoxanthin was found to induce cellular DNA fragmentation/internucleosomal DNA degradation by activation of endogenous nucleases in a dose dependent manner unlike β-carotene and astaxanthin. The authors have hypothesized that since fucoxanthin is converted to fucoxanthinol prior to uptake and also fucoxanthinol shows greater inhibition of growth, the superior anti-proliferative effect of fucoxanthin may be due to its metabolites [27]. DNA fragmentation typical of apoptotic cells was observed in NSCLC-N6 (human non-small cell bronchopulmonary carcinoma) cells treated with fucoxanthin along with typical morphological changes such as rounding up, reduction in cell volume, chromatin condensation, nuclei fragmentation, and formation of apoptotic bodies [36]. However, in the same study, no apoptosis was observed in SRA (human lens epithelial cells) indicating the specific action of fucoxanthin against carcinogenic cells. 

The results obtained by Konishi *et al*. [50] show the dose and time dependent anti-proliferative effect of fucoxanthinol and fucoxanthin in HL-60, MCF-7 (breast cancer), and Caco-2 cells. In their studies, they found the anti-proliferative effects of fucoxanthinol to be greater than fucoxanthin. Their experiments involved studying the effect of metabolites like fucoxanthinol and halocynthiaxanthin and comparing it with fucoxanthin. They have concluded that both the metabolites showed superior anti-proliferative activity and have speculated that one of the factors that may contribute to this may be the presence of hydroxyl functional group in place of the acetyl group of fucoxanthin, a feature common to both metabolites, in addition to unique structures such as 5,6-epoxide, acetylenic and allenic bonds. Zhang *et al*. [51] have reported the anti-proliferative effect of fucoxanthin on EJ-1 (urinary bladder cancer) cells. Apoptosis characterized by condensed chromatin, nuclear fragmentation, and apoptotic bodies in addition to the DNA ladder was observed.

Expression of v-FLIP and v-cyclin was inhibited and may be responsible for growth inhibition and apoptosis observed with fucoxanthin treatment [34]. The authors have suggested that the effective concentration for the induction of apoptosis is different in different cell lines and fucoxanthinol exhibits more potent activity as compared to fucoxanthin. GADD153 known to be associated with apoptosis was also induced by fucoxanthin in a study [35].

### 2.6. Metastasis

Metastasis is the stage of cancer at which tumor cells acquire the advantageous characteristics that allows them to escape from the primary tumor and migrate to surrounding and distant organs and tissues. Metastasis involves the interaction of the tumor cells with numerous factors and cell components including matrix metalloproteinases (MMPs). MMPs are thought to assist tumor cells in metastasis and their enhanced levels have been associated with extra-cellular matrix degradation and cancer cell invasion [52]. Chung *et al*. [53] have studied the effect of fucoxanthin in B16-F10 cells (metastatic murine melanoma) MMP-2 and MMP-9. These MMPs are expressed in cancer cells and degrade type IV collagen during cancer invasion. Fucoxanthin treatment resulted in decreased expression and secretion levels of MMP-9. Moreover; the numbers of invaded B16-F10 cells were also decreased. In addition to MMPs; they have also studied CD44 and CXR4; a cell surface glycoprotein and CXC chemokine receptor respectively; that are also known to be up-regulated in cancer metastasis. Fucoxanthin was found to reduce the mRNA expression of CD44 and CXCR4 in a dose dependent manner. Fucoxanthin was also shown to decrease cell motility of melanoma cells which was ascertained by reduction in stress fiber and lamellipodia formation that are important in cell migration. Hence; by suppressing cancer cell motility and invasion factors; fucoxanthin may be valuable in preventing cancer cell metastasis. 

## 3. Molecules and Mechanisms Related to Apoptosis

### 3.1. Bcl-2 Proteins

The family of Bcl-2 proteins has anti-apoptotic and pro-apoptotic members. Anti-apoptotic Bcl-2 family proteins include Bcl-2, Bcl-x_L_, A1, Bcl-w, and Boo, while the pro-apoptotic members include Bax and Bak, Bok, Bcl-xs, Bim, Bad, Bid, Bik, Bmf, Puma, Noxa, and Hrk [54,55]. Several researchers who have tried to elucidate the mechanism underlying the anti-proliferative effect of fucoxanthin have studied the Bcl-2 family of proteins. Many studies have reported the down regulation of Bcl-2 expression in HL-60, Caco-2 cells [12,27,50]. Nakazawa *et al*. [56] observed down-regulation of Bcl-2 proteins, which was associated with the apoptosis in their study, which compared the effects of cis and trans forms of fucoxanthin. Their results have suggested that while the uptake of trans form was higher in the cells (HL-60 human leukemia, Caco-2 colon cancer, PC-3 and LNCap prostate), the apoptosis effect of the cis forms was higher. They have attributed this difference to the stearic hindrances arising from their different chemical structures. As the ratio of pro and anti-apoptotic Bcl-2 members is an important determinant of cell viability and apoptosis, Liu *et al*. [57] studied these factors and reported an increase in Bax/Bcl-2 mRNA expression when a combination of cisplatin and fucoxanthin were administered. Down-regulation of Bcl-x_L_ and XIAP by fucoxanthin was observed by Yamamoto *et al*. [31] and Kim *et al*. [15], while Ishikawa *et al*. [14] reported decreased expression of Bcl-2 and XIAP, and Kim *et al*. [23] observed decreased expression of Bcl-x_L_, Kotake-Nara *et al*. [35] on the other hand have speculated that as Bax (pro-apoptotic) and Bcl-2 (anti-apoptotic) were down-regulated by fucoxanthin while Bcl-x_L_ was unaltered, unlike other apoptosis-inducing agents that modulate the ratios of the pro- and anti-apoptotic proteins, fucoxanthin may operate through a different pathway.

### 3.2. The Caspase Pathway

The caspases are cysteine proteases that control apoptosis. The extrinsic pathway involves the tumor necrosis factor and activates the caspases 8 and 10 while the intrinsic pathway involves the mitochondria and release of cytochrome c from damaged mitochondria, activating Caspase-9, which is an initiator and in turn can cleave and activate the effector Caspases such as Caspases-3, -6, and -7. These two Caspase pathways, intrinsic and extrinsic, can result in apoptosis. The intrinsic pathway involving the mitochondria and caspases-3, -6, -7, and -9 are controlled by the Bcl-2 protein family [55]. The activation of one or more members of the caspase pathways by fucoxanthin has been reported by several researchers. Wang *et al*. [12], Ganesan *et al*. [26], Zhang *et al*. [51] have observed the increased expression of caspase-3 on exposure to fucoxanthin. Fucoxanthin and fucoxanthinol treatments resulted in production of cleaved products and thus activation of PARP, caspase-3, -8, and -9 demonstrating that caspase activation plays a role in the apoptosis observed [31]. Fucoxanthinol activated caspase pathways with cleavage of caspase-3, -8, -9, and PARP [14]. Expression of caspase-3 and -9 was increased by fucoxanthin. PARP, a substrate of caspases was detected in its intact and cleaved form, indicating apoptosis [15]. Fucoxanthin was found to activate the caspase pathway with the cleavage of caspase-3 and PARP and increased activities of caspase -8 and -9 as well [22]. The results suggested that fucoxanthin accumulates in the mitochondrial membranes causing a reduction in the membrane potential and release of cytochrome c from mitochondria to the cytosol, followed by caspase-9 and caspase-3 activation, leading to the apoptotic effects of fucoxanthin in the leukemia cell lines. In a separate study where the authors compared the effect of fucoxanthin and neoxanthin in PC-3 prostate cells, the down regulation of procaspase-3 and PARP and the increased active fragment of caspase-3 and cleaved PARP indicated caspase-3 dependent apoptosis by fucoxanthin [35].

### 3.3. MAPK and GADD45

The MAPK family or the mitogen activated protein kinase includes four well characterized sub-groups: (1) ERK1 and ERK2 that are extracellular signal kinases; (2) JNK1, JNK2, and JNK3 with the c-Jun NH_2_ terminal kinases, also called as stress-activated protein kinases (SAPK); (3) p38 enzymes including p38α, p38β, p38γ, p38δ, and the recently identified; (4) ERK5. ERK1 and ERK2 regulate cell processes like mitosis, meiosis, post mitotic functions, and are responsible for proliferation, cell division, differentiation, development and survival. STAT (signal transducers and activators of transcription) proteins such as Stat3 are substrates that are phosphorylated by ERK and are activators of transcription. The JNK/SAPKs are activated by conditions such as oxidative stress and result in programmed cell death or apoptosis, growth and cell cycle arrest as well as inflammation and tumorigenesis and cell survival under certain conditions. C-Jun is a component of the AP-1 complex that is an important regulator of gene function and is activated by environmental stress, radiation and growth factors. The p38 MAPKs control the expression of several cytokines and are involved in the immune response mechanism in addition to cell motility, apoptosis, chromatin remodeling, and osmoregulation [58,59]. Gadd45 proteins include the subtypes GADD45A, GADD45B, GADD45G, and are involved in cell cycle arrest at the G2/M and G1 stages (depending on the interactions), DNA repair, cell survival and apoptosis and are known to interact with the members of the MAPK family. In addition, GADD45A and GADD45G are repressed by the activated members of the NFκB family in various types of cancer [60,61]. 

Fucoxanthin was found to attenuate cisplatin induced phosphorylation of ERK, p38, and P13K/AKT (phosphatidylinositol 3 kinase family) in the studies carried out by Liu *et al*. [57]. Their experiments with specific inhibitors also revealed that fucoxanthin may inhibit ERCC1 mRNA expression via the ERK and P13K/AKT pathway while thymidine phosphorylase mRNA expression may be inhibited through the p38 pathway. Ishikawa *et al*. [14] have observed decreased AP-1 DNA binding activity and JunD expression indicating the inactivation of AP-1 by depletion of JunD. Their results indicated that up-regulation of the GADD45α observed was independent of p53. 

In a separate study, Satomi and Nishino [43] found that fucoxanthin activated the p38 and ERK1/2 MAPKs in HepG2 cells and SAPK/JNK pathways were activated in DU145 cells, indicating that SAPK/JNK are upstream activators of GADD45 expression. Thus, each MAPK and each GADD45 subtype (a and b) play different roles in cell cycle progression. They have concluded that fucoxanthin induces cell arrest by a GADD45A dependent pathway and the GADD45A expression and G1 arrest are negatively regulated by p38 MAPK in HepG2 cells and positively regulated by the SAPK/JNK pathway in DU145 cells. In another study, CYP1A1 gene expression and other cell cycle and growth related genes such as GADD45A, GADD45B, PIM1 and IFRDI were induced by fucoxanthin as well as the expression of other cell cycle related genes such as p21, p27 and c-myc [32]. Further, Satomi [44] has reported the role of fucoxanthin on the MAPKs (extracellular signal-regulated kinases ERK1/2, ERK5, p38 MAPK kinases, c-Jun *N*-terminal kinases (SAPK/JNK)) and its association with the GADD45 activation for cell growth arrest in LNCap cells (prostate cancer). GADD45A may be implicated in the G1 arrest observed in the study. While the GADD45A gene was enhanced, GADD45B gene expression was unaffected after fucoxanthin treatment. With respect to the MAPK family, SAPK/JNK was increased, phosphorylation of ERK 1/2 was reduced and phosphorylation of p38 was unaffected. While it is suggested that MAPKs including SAPK/JNK induce GADD45A in a p53 dependent or independent manner, the author has suggested a p53 independent mechanism in the present study. In addition, inhibition of the SAPK/JNK pathway reduced GADD45A induction while inhibition of ERK 1/2 and p38 pathways stimulated GADD45A induction. The author has suggested that each MAPK plays a different role in GADD45A induction and G1 arrest by fucoxanthin based on the negative regulation of p38 MAPK resulting in increased GADD45A expression along with other observations in prostate cancer cells. Contrasting results obtained for ERK 1/2 MAPK in LNCap cells and in DU145 cells earlier indicate that GADD45A may not be the only factor responsible for the G1 cell arrest observed in that study. Thus the growth inhibitory effect exhibited by fucoxanthin may in part be due to a GADD45A-dependent pathway and the enhanced GADD45A expression and G1 arrest are positive regulated by SAPK/JNK in prostate cancer cells.

Yu *et al*. [47] have observed the down-regulation of STAT3 at mRNA and protein levels, which indicated the inhibition of the JAK/STAT pathway by fucoxanthin as the JAK are known to activate the STAT members. Further, Wang *et al*. [12] reported that the STAT3 and p-STAT3 (phosphorylated STAT3) was down regulated by fucoxanthin. EGFR (epidermal growth factor receptor) expression, which is often dysregulated in many human cancers, was decreased with fucoxanthin. They have concluded that down regulation of STAT3/EGFR was involved in the anti tumor and apoptosis inducing effects of fucoxanthin.

### 3.4. NFκB

The nuclear factor kappa B (NF-κB) is a family of closely related transcription factors that are held in the cytoplasm in the inactive form by their interaction with the inhibitor of κB (IκB). IκBs include IκBα, IκBβ, IκBε, and BCL-3. The phosphorylation of IκB results in activation of NFκB and its translocation to the nucleus, followed by induction of target genes and the resulting effects. NF-κB may be activated by many cytokines, growth factors and their receptors, tyrosine kinases, tumor necrosis factor receptor families, other signaling pathways, such as Ras/MAPK and PI3K/Akt. NF-κB promotes resistance to apoptosis and may also exhibit pro-apoptotic properties. NF-κB inhibits p53-induced apoptosis by up-regulating anti-apoptotic genes, and decreasing p53 levels. As NF-kB is associated with several tumor/cancer related processes, such as its activation by pro-inflammatory cytokines and its ability to induce cell proliferation and anti-apoptotic gene expression, as well as induction of angiogenesis, it is often considered as a hallmark of cancer [62,63]. 

Cisplatin has the potential to bind to the DNA molecules, forming platinum-DNA adducts which interfere with transcription and replication of the DNA, resulting in cell death [64,65]. Several mechanisms responsible for resistance to cisplatin are known. Elevated mRNA levels of excision repair cross-complementation group 1 (ERCC1) are reported in clinical resistance to platinum-based chemotherapy for various cancers. NFκB induces cell proliferation, metastasis, suppression of apoptosis, oncogenesis, and cancer therapy resistance. NFκB induces anti-apoptotic proteins and suppresses pro-apoptotic genes and thus inhibits apoptosis. Cisplatin and several other cancer drugs are shown to induce NFκB translocation and activation, resulting in drug resistance. In addition, thymidine phosphorylase (TP) expression is higher in tumors, and results in their enhanced resistance to apoptosis. Mise and Yasumoto [30] found that that the antioxidative potential of fucoxanthin did not decrease the cytotoxicity of the platinum anti-cancer drug, cisplatin. In another study, Liu *et al*. [57] have also reported the similar effectiveness of fucoxanthin with cisplatin, in HepG2 cells. The results of their study clearly indicated an enhanced anti-proliferative effect of cisplatin when used in combination with fucoxanthin. NFκB binding was decreased and restoration of IκB by inhibition of phosphorylation was increased. Their results involving an NFκB inhibitor suggests an inhibition of the NFκB pathway when the combination is administered as compared to cisplatin alone. While cisplatin resulted in increased expression of ERCC1 and TP, combined treatment with fucoxanthin resulted in their inhibition. In another study, fucoxanthin and fucoxanthinol reduced the phosphorylation of IKKβ and IκBα and levels of IKKα, IKKβ, and IKKγ (indicating Hsp90 chaperone inhibition) [31]. Different genes showed different susceptibility to the fucoxanthin treatment and were associated with the down regulation of DNA-binding activities of NFκB, pIκBα, and increased IκBα [14].

### 3.5. CYP3A4 Enzyme

Due to their wide range of substrate selectivity CYP3A enzymes play a major role in metabolism and are of special relevance in the metabolism of clinically used drugs [66]. The cytochrome P 450 3A4 (CYP3A4) is the most abundant of the P450 isoforms and is found in the liver and intestines of humans. Activation of pregnane X Receptor (PXR) results in the induction of CYP3A4. Cytochrome P450 enzymes are regulated by both the PXR and CAR (constitutive androstane receptor) pathways [67]. PXR is known to act as a xenobiotic sensor and can prevent intracellular accumulation of drugs by activation of cytochrome P450 and multiple drug resistance 1 (MDR 1). PXR activation has an anti-apoptotic role and PXR antagonists can decrease cell proliferation and interfere with cancer drug resistance [68,69]. Liu *et al*. [70] have examined the possible role of fucoxanthin as an adjuvant to prevent or overcome rifampin induced drug resistance in HepG2 and LS174T cells. In their study, decreased basal CYP3A4 enzyme activity, CYP3A4 mRNA expression, CYP3A4 protein expression, CYP3A4 promoter activity through CAR and decreased PXR and SRC-1(co-activator of PXR) interaction in fucoxanthin treated group and with co-incubation of fucoxanthin with rifampin was observed. Their results indicate that fucoxanthin may play an important role as not only a chemotherapeutic agent, but also in assisting other cancer drugs by attenuating the prevailing drug resistance. Satomi and Nishino [32] have observed induction of the CYP1A1 gene expression as a result of fucoxanthin treatment in prostate cancer cell lines.

### 3.6. Gap Junctional Intracellular Communication/Connexin Genes

Gap junctional intracellular communication (GJIC) is a mechanism for intercellular cell communication and operates at sites of cell adhesion where plasma membranes of cells can be connected by buried paired channels. Thus, GJIC regulates the communication between cells of tissues of an organ, allowing for direct communication between the cytoplasm of cells without transit through the extracellular space, making it possible for the cells to achieve a common and integrated target/metabolic activity [71]. Gap junctions in vertebrates are composed of the connexin family of proteins, which are about 20 in number in humans [72]. Loss and impairment of GJIC has been associated with pathologies such as cancer, heart and skin diseases, cataracts, hereditary deafness, and some forms of neuropathy [73]. The enhancement of GJIC in cancer on treatment with several carotenoids such as β-carotene, canthaxanthin, lutein, α-carotene, lycopene astaxanthin, has been reported [74,75,76]. Liu *et al*. [29] have attempted to elucidate the mechanism of the anti-proliferative action of fucoxanthin by studying the GJIC, expression of connexin genes and DNA damage. The GJIC and expression of connexin genes (Cx 32 and Cx 43) was improved at both protein and mRNA levels in the SK-Hep-1 cells in a concentration dependent manner by fucoxanthin while the BNL CL.2 cells were unaffected. Their results indicated that fucoxanthin can regulate pathways, such as MAPK and PI3 kinase/Akt cascades, which are known to have a negative effect on GJIC. They have concluded that fucoxanthin exhibits anti-proliferative activity in SK-Hep-1 cells along with enhanced expression of connexin genes and GJIC. Satomi and Nishino [32] have also observed an increase in expression of connexin 43 and AP-1.

### 3.7. Expression of N-Myc Oncogene, Survivin, and Angiogenic Activity

N-Myc oncogene, known to be over expressed in neuroblastoma, was reduced by fucoxanthin treatment in GOTO neuroblastoma cell line and this effect was found to be reversible when fucoxanthin was removed from the media [10]. Survivin is known to be prominently expressed in many common human cancers. Yu *et al*. [47] studied the role of JAK/STAT pathway and have speculated that other pathways may inhibit the expression of survivin. Further, the expression of survivin and VEGF (vascular endothelial growth factor; induced by STAT3) positive cells was down regulated by fucoxanthin in the study of Wang *et al*. [12]. Yamamoto *et al*. [31] and Ishikawa *et al*. [14] also observed the down-regulation of survivin.

Sugawara *et al*. [77] have reported the anti-angiogenic activity of fucoxanthin and postulated that this may be another protective effect of fucoxanthin in pathologies such as cancer. They have observed the reduced tube length of HUVEC (human umbilical vein endothelial cells) cells and the inhibition of their proliferation by fucoxanthin but have observed no effect on their migration. Fucoxanthin and fucoxanthinol were found to suppress development of blood vessel like structures in embryonic stem cell derived embyoid bodies and outgrowth of micro-vessels. In addition they have hypothesized that antioxidant activity of fucoxanthin may be involved in its anti-angiogenic effect as ROS are known to stimulate angiogenesis. 

## 4. Conclusions

Fucoxanthin influences a multitude of molecular and cellular processes. It exerts strong effects on cancer cells and shows synergistic activity in combination with established cytotoxic drugs. This raises the possibility that it could become an interesting anti-cancer compound in various types of cancer.
